# Measuring event concentration in empirical networks with different types of degree distributions

**DOI:** 10.1371/journal.pone.0241790

**Published:** 2020-12-02

**Authors:** Juan Campos, Jorge Finke

**Affiliations:** 1 Sports Models Division, Genius Sports, Medellín, Colombia; 2 Department of Electrical Engineering and Computer Science, Pontificia Universidad Javeriana, Cali, Colombia; Unviersity of Burgundy, FRANCE

## Abstract

Measuring event concentration often involves identifying clusters of events at various scales of resolution and across different regions. In the context of a city, for example, clusters may be characterized by the proximity of events in the metric space. However, events may also occur over urban structures such as public transportation and infrastructure systems, which are naturally represented as networks. Our work provides a theoretical framework to determine whether events distributed over a set of interconnected nodes are concentrated on a particular subset. Our main analysis shows how the proposed or any other measure of event concentration on a network must explicitly take into account its degree distribution. We apply the framework to measure event concentration (i) on a street network (i.e., approximated as a regular network where events represent criminal activities); and (ii) on a social network (i.e., a power law network where events represent users who are dissatisfied after purchasing the same product).

## Introduction

Consider a non-uniformly distribution of events over different regions. Past efforts to explain the mechanisms through which some regions reveal a high concentration of events (i.e., form hotspots) range from agent-based [1], game theoretic [2, 3], reaction-diffusion [4], and predator-prey [5] modeling. Generally, these approaches account for the relative location of events on the metric space, and use kernel density techniques to identify and recreate hotspots [6, 7]. For a number of scenarios, however, the concentration of events can be better captured by their distribution over a set of interconnected nodes [8, 9].

The work in [8] adapts the main idea behind kernel density techniques [6, 7] to identify hotspots on networks. In particular, a hotspot indicates a subnetwork that contains the maximum number of events on the smallest total path length. The authors consider two types of networks, namely, binary trees and regular networks. To compute the optimal subnetwork for binary trees, they introduce an algorithm that identifies hotspots based on dynamic programming. For regular networks, identifying hotspots requires that all possible subnetworks be evaluated, which becomes computationally costly for networks of large size. A second shortcoming of the work in [8] is that the approach does not extend to networks with more realistic topologies. In most cases, empirical networks exhibit degree distributions under which the nodes connect to different numbers of neighbors (ranging orders of magnitude in degree values, e.g., for networks with power law degree distributions).

The work in [9] introduces an alternative approach, which evaluates the concentration of events on networks with different types of degree distributions (namely, networks with with regular, Poisson, and power law degree distributions) based on Voronoi diagrams [10]. Nodes that are associated with the occurrence of a certain number of events are marked as *generator nodes*. Voronoi cells are then defined according the geodesic distances from generator nodes to all other nodes of the network. The measure of concentration of events that the authors propose builds on a key property of Voronoi diagrams: groups of small, adjacent cells (created by generator nodes) correspond to subnetworks with a high event concentration.

Simulation results in [9] illustrate that evaluating event concentration in networks depends on their degree distribution. This paper extends the work in [9] in two ways. First, we provide the mathematical foundation to characterize the theoretical distribution of the sizes of the Voronoi cells when events are located uniformly at random over a network with an arbitrary distribution. Second, we use the resulting distribution and apply the criterion in [9] to measure event concentrations in empirical networks. In particular, we consider events that represent (i) criminal activities on a street network (approximated as a regular network), and (ii) dissatisfied users on a social network (a power law network). Our results illustrate the importance of understanding the relationship between the degree distribution and the dispersion of events on a network in order to identify and recreate the formation of hotspots.

The remaining sections are organized as follows. Section 2 introduces some notation and preliminaries. Section 3 presents the proposed framework for measuring event concentration, and introduces the criterion for detecting hotspots based on a summary statistic derived from the proposed framework. Section 4 applies the criterion to the two empirical networks and compares the outcome to that of detecting hotspots by the proximity of events in the metric space. Finally, Section 5 draws some conclusions and future research directions.

## Preliminaries

Consider an undirected network *G* = (*V*, *E*), where *V* = {*v*_1_, ⋯, *v*_*n*_} denotes the set of nodes and *E* ⊆ *V* × *V* the set of edges. The geodesic distance between nodes *v*_*i*_ and *v*_*j*_ is denoted by *ρ*(*v*_*i*_, *v*_*j*_). We borrow the definition of a Voronoi diagram of a network from [11].

**Definition 1**. Suppose that there exists a subset of nodes marked as generator nodes. This subset is denoted by *U* = {*u*_1_, ⋯, *u*_*m*_} ⊆ *V*. The Voronoi diagram of *G* = (*V*, *E*) associated to the nodes belonging to *U* is a partition {*V*(*u*_1_), ⋯, *V*(*u*_*m*_)} of *V*, such that:

- If *v*_*i*_ ∈ *V*(*u*_*s*_), then *ρ*(*v*_*i*_, *u*_*s*_) ≤ *ρ*(*v*_*i*_, *u*_*s*′_) for all *s*′ ∈ {1, ⋯, *m*}.
- If *ρ*(*v*_*i*_, *u*_*s*_) = *ρ*(*v*_*i*_, *u*_*s*′_), then node *v*_*i*_ is equally likely to be assigned to *V*(*u*_*s*_) or *V*(*u*_*s*′_).

Let *u*_*i*_ represent a generator node which is associated to the occurrence of at least a certain number of events ε∈Z,ε≥0. A generator node may capture, for example, an intersection on a representation of a street network where *ε* or more criminal activities occur. Regular nodes, on the other hand, represent intersections at which less than *ε* events occurs. Such nodes belong to the set *U*^*c*^ = *V* − *U*. Note that the generator node associated to cell *V*(*u*_*s*_) is denoted by *u*_*s*_ and a cell refers to an element of the Voronoi partition. Note also that any cell *V*(*u*_*s*_) contains one generator node. Finally, note that if the network *G* is a connected network, then any regular node *v*_*i*_ ∈ *V* belongs to some cell.

Based on Definition 1, *n*_*s*_ = |*V*(*u*_*s*_)| ≥ 1 denotes the size of cell *V*(*u*_*s*_). The distribution of *n*_*s*_ for all *u*_*s*_ ∈ *U* for *G*, that is, the distribution of the sizes of all cells determines whether events on *G* = (*V*, *E*) are uniformly distributed. Deviations from a uniform distribution evidence a concentration of events that results from a non-uniform allocation.

**Fig 1:**
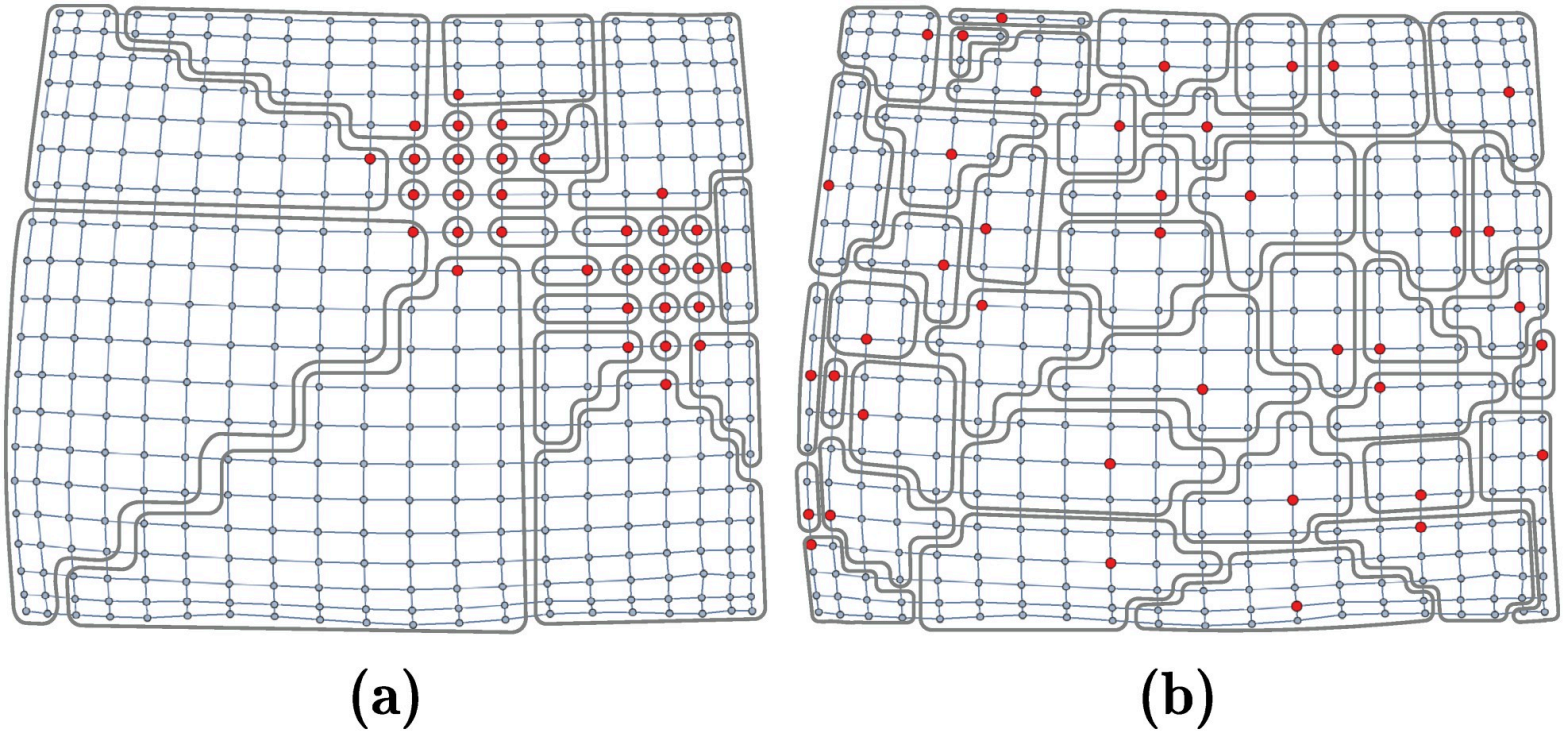
Voronoi cells. Resulting Voronoi cells when generator nodes (defined by the occurrence of at least *ε* events) are (a) concentrated, and (b) distributed uniformly at random. For this scenario, a node is labelled as a regular node only if no event occurs at that node (*ε* = 1). Otherwise, if one or more events occur, the node is marked as a generator node. In general, the event threshold *ε* defines the minimum number of events that must occur for a node to be marked as a generator node.

**Fig 2:**
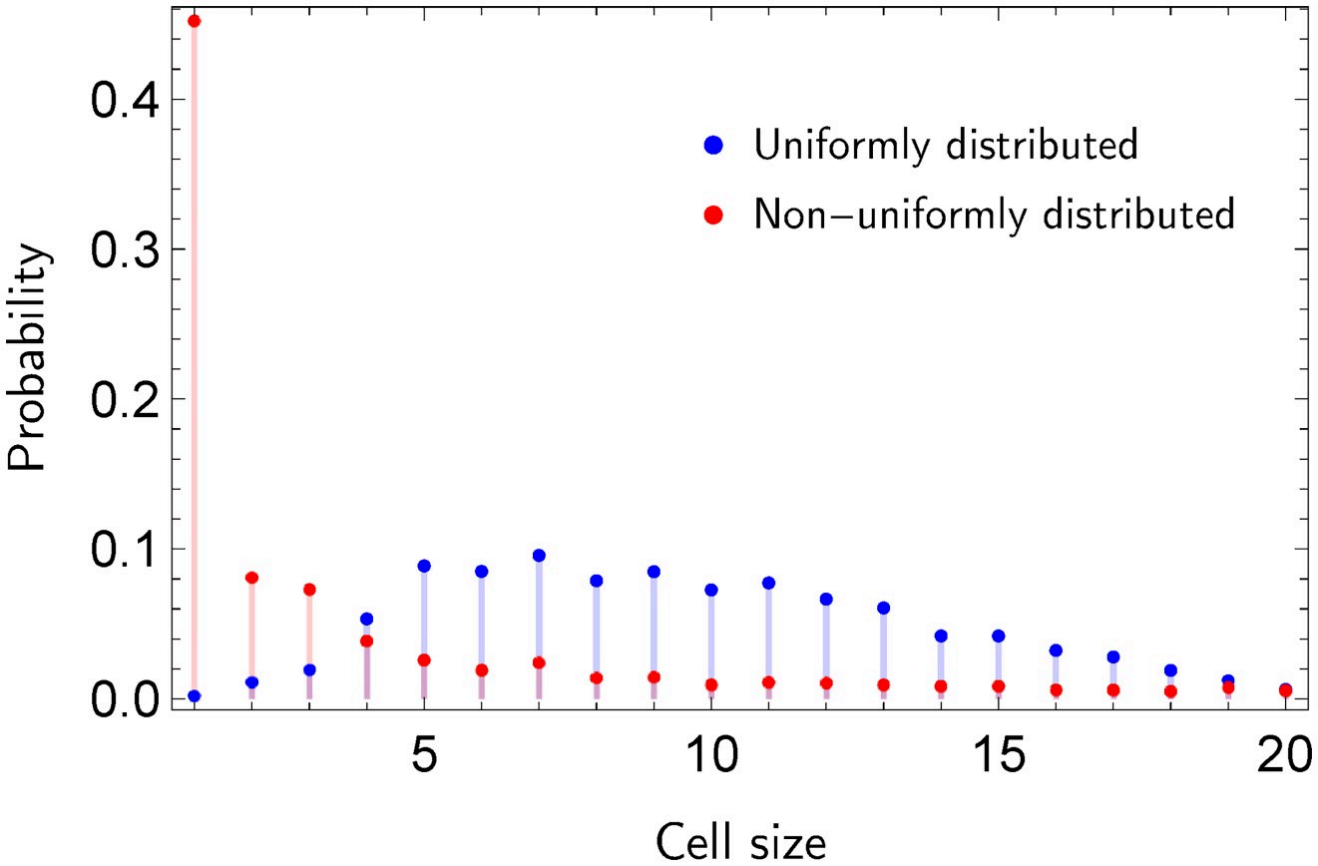
Probability distributions of the sizes of the Voronoi cells.

Fig 1 shows the Voronoi cells in a scenario with two particular cases. Note that in Fig 1(a) the non-uniform allocation of the generator nodes yields relative small geodesic distances between them. In comparison to Fig 1(b), where generator nodes (events) are uniformly distributed, most cells of the Voronoi diagram in Fig 1(a) contain a small number of regular nodes. Fig 2 depicts the probability mass function (pmf) of the sizes of the cells for uniform and non-uniform event allocations.

Next, consider a randomly selected node *v*, with degree *d*_*v*_ = *d*. Let $N_{\delta }^{v}=\left\{v^{\prime }\in V:\rho \left(v,v^{\prime }\right)=\delta \right\}$ represent the neighborhood of nodes located at a distance *δ* from node *v*. Furthermore, let *D* denote a random variable that represents the degree of a randomly selected node. And finally, let $D_{d}^{\delta }$ denote a random variable that represents the degree of a randomly selected node in $N_{\delta }^{v}$.

To derive the pmf of the sizes of the Voronoi cells resulting from a uniform distribution of events, consider the following assumptions. Suppose that the degree distribution (i.e., the pmf of *D*) and the conditional degree distribution (i.e., the pmf of $D_{d}^{1}$) are known [12]. Furthermore, suppose that the pmf of $D_{d}^{\delta }$ for *δ* ≥ 2 can be approximated as

$$\begin{array}{c} P\left[D_{d}^{\delta }=d_{i}\right]\approx \frac{nP\left[D=d_{i}\right]\;d_{i}}{2|E|}=\frac{P\left[D=d_{i}\right]\;d_{i}}{\bar{k}} \end{array}$$ (1)

where $\bar{k}$ is the average degree of all nodes of the network.

Under the above assumptions, we are now able to introduce a framework that defines the pmf of the sizes of Voronoi cells when events are distributed uniformly at random. The notation for the framework is summarized in Table 1.

**Table 1:** Notation for determining event concentration on networks.

Notation	Meaning
*X*	Size of the cell of a randomly selected generator node
*X*_*d*_	Size of the cell of a randomly selected generator node *u* with *d*_*u*_ = *d*
$X_{d}^{1}$	Number of nodes in $V\left(u\right)\cap N_{1}^{u}$, where *d*_*u*_ = *d*
$X_{d}^{2i}$	Number of nodes in $V\left(u\right)\cap N_{2}^{u}$, *d*_*u*_ = *d*, that are themselves neighbors of node *v*_*i*_ in $N_{1}^{u}$
*D*	Degree of a randomly selected node
*D*_*g*_	Degree of a randomly selected generator node
$D_{d}^{1}$	Degree of a randomly selected node in $N_{1}^{u}$
$D_{d}^{2}$	Degree of a randomly selected node from $N_{2}^{u}$, that is itself a neighbor of a node in $V\left(u\right)\cap N_{1}^{u}$

## Framework and hotspot detection criterion

Let *D*_*g*_ denote a random variable that represents the degree of a randomly selected generator node. Furthermore, the proportion of nodes that are generator nodes is denoted by *p* = *m*/*n*, 0 < *p* < 1. Consider a randomly selected generator node, denoted node *u*. For convenience, we will write *N*_*δ*_ to denote $N_{\delta }^{u}$.

**Assumption 1**. Suppose that

1. The degree distribution of the generator nodes resembles the degree distribution of all nodes of *G*.
2. If *v* ∈ *V*(*u*), *v* ≠ *u*, then *v* ∈ *N*_1_ ∪ *N*_2_.
3. The local clustering coefficient of node *u* is negligible (less than 0.1).
4. Nodes in *N*_2_ have a single neighbor in *N*_1_.

**Fig 3:**
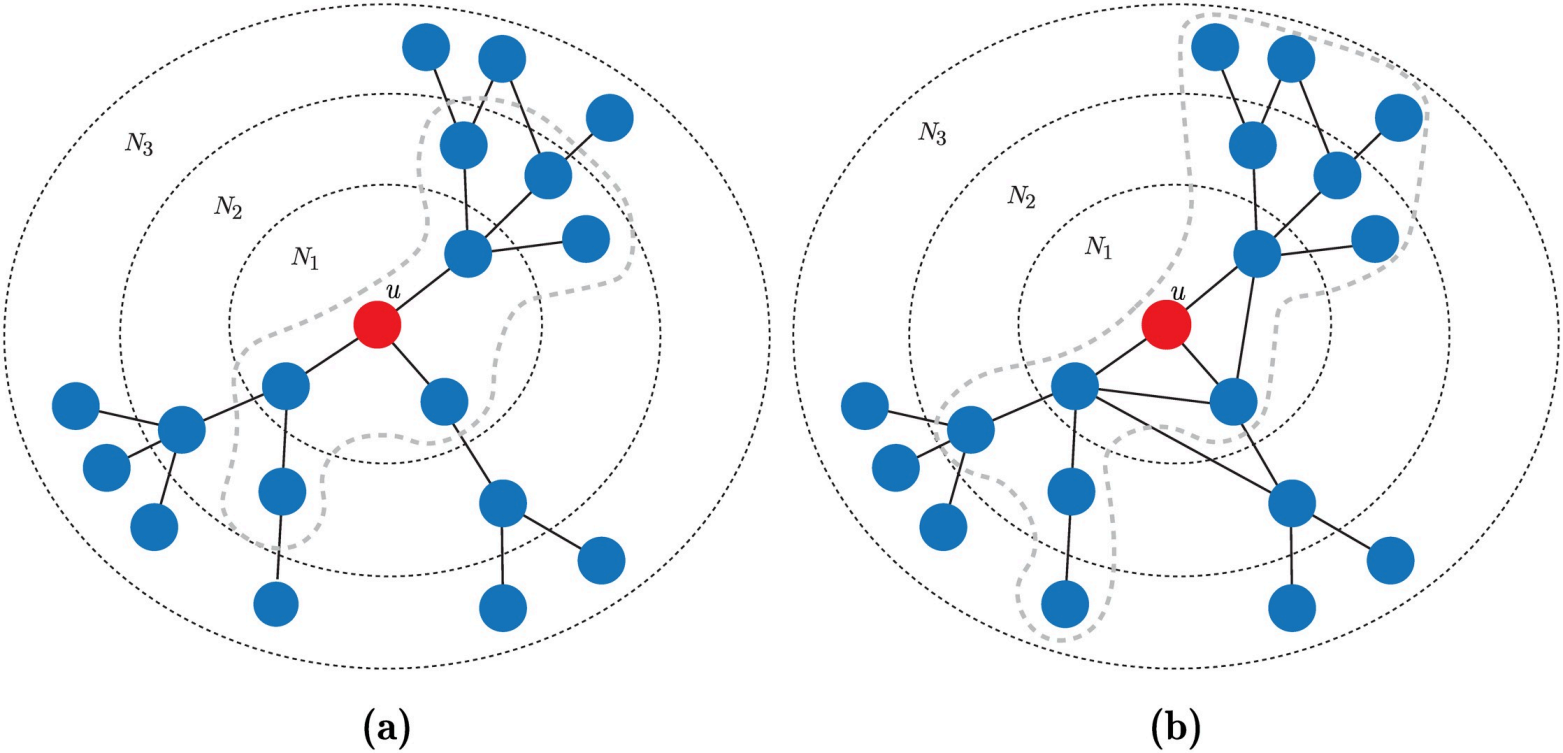
Illustration of the conditions of Assumption 1 (except the first condition). The boundary indicates the cell *V*(*u*). (a) Scenario where conditions 2, 3 and 4 are satisfied, and (b) scenario where neither condition is satisfied.

Fig 3 illustrates conditions 2-4 of Assumption 1. These conditions are satisfied for Fig 3(a) but not for Fig 3(b).

The degree of generator node *u* is denoted by *d*_*u*_ = *d*. Note that the random variable $D_{d}^{\delta }$ represents the degree of a randomly selected node in *N*_*δ*_. Let *X*_*d*_ denote a random variable that represents the size of *V*(*u*). Moreover, let $X_{d}^{1}$ denote a random variable that represents the number of nodes in *V*(*u*) ∩ *N*_1_. Similarly, let $X_{d}^{2i}$ denote a random variable that represents the number of nodes in *V*(*u*) ∩ *N*_2_, that are themselves neighbors of node *v*_*i*_, located in *V*(*u*) ∩ *N*_1_. Note that $X_{d}^{2i}$ and $X_{d}^{2j}$ are independent and identically distributed random variables.

Note that if conditions 3 and 4 of Assumption 1 are satisfied, then

$$\begin{array}{c} X_{d}=(\sum_{i=1}^{X_{d}^{1}}X_{d}^{2i})+X_{d}^{1}+1 \end{array}$$ (2)

The first term in Eq (2) characterizes the number of nodes in *N*_2_, which depends on the realization of $X_{d}^{1}$. The term $X_{d}^{1}+1$ characterizes the number of nodes in *N*_1_ plus the generator node. Let $F_{1d}\left(x\right)=P\left[X_{d}^{1}=x\right]$ and $F_{2d}\left(x\right)=P\left[X_{d}^{2i}=x\right]$ represent the pmfs of $X_{d}^{1}$ and $X_{d}^{2i}$. Moreover, let *k*_*m*_ represent the minimum degree of all nodes. The following theorem characterizes *F*_1*d*_(*x*).

**Theorem 1**. The pmf of $X_{d}^{1}$ is given by

$$\begin{array}{c} F_{1d}\left(x\right)=\sum_{i=0}^{d}P\left[R_{d}^{1}=i\right]P_{1}\left(x,i,P\left[W_{d}^{1}=1\right]\right) \end{array}$$ (3)

where

$$\begin{array}{cc} P[R_{d}^{1}=x] & =P_{1}\left(x,d,1-p\right) \end{array}$$ (4)

$$\begin{array}{cc} P[Z_{d}^{1}=x] & =\sum_{d_{i}=k_{m}}^{n}P\left[D_{d}^{1}=d_{i}\right]P_{1}(x,d_{i}-1,p) \end{array}$$ (5)

$$\begin{array}{cc} P[W_{d}^{1}=x] & =\sum_{i=0}^{n}P\left[Z_{d}^{1}=i\right]P_{2}(x,\frac{1}{1+i}) \end{array}$$ (6)

$$\begin{array}{cc} P_{1}\left(x,d,q\right) & =\left(\begin{array}{c} d\\ x \end{array}\right)q^{x}{\left(1-q\right)}^{d-x} \end{array}$$ (7)

$$\begin{array}{cc} P_{2}\left(x,q\right) & =q^{x}{\left(1-q\right)}^{1-x} \end{array}$$ (8)

Note that *F*_1*d*_(*x*) can be obtained if *p* and the pmf of $D_{d}^{1}$ are known. The proof of Theorem 1 and all other theorems can be found in appendices. Next, Theorem 2 characterizes *F*_2*d*_(*x*).

**Theorem 2**. The pmf of $X_{d}^{2i}$ is given by

$$\begin{array}{c} F_{2d}\left(x\right)=\sum_{i=0}^{n}P\left[Y_{d}^{2}=i\right]P_{1}\left(x,i,P\left[W_{d}^{2}=1\right]\right) \end{array}$$ (9)

where

$$\begin{array}{cc} P[R_{d}^{2}=x] & =\frac{\sum_{d_{j}=i+1}^{n}P\left[D_{d}^{1}=d_{j}\right]P_{1}\left(x,d_{j}-1,1-p\right)(\frac{1}{d_{j}-i})}{\sum_{k=0}^{n}\sum_{d_{j}=k+1}^{n}P\left[D_{d}^{1}=d_{j}\right]P_{1}\left(k,d_{j}-1,1-p\right)(\frac{1}{d_{j}-k})}, \end{array}$$ (10)

$$\begin{array}{cc} P[G_{d}^{2}=x] & \approx \sum_{d_{i}=k_{m}}^{n}\frac{P\left[D=d_{i}\right]\;d_{i}}{\bar{k}}P_{1}\left(x,d_{i}-1,p\right) \end{array}$$ (11)

$$\begin{array}{cc} P[Y_{d}^{2}=x] & =\sum_{i=0}^{n}P\left[R_{d}^{2}=i\right]P_{1}\left(x,i,P\left[G_{d}^{2}=0\right]\right) \end{array}$$ (12)

$$\begin{array}{cc} P[{\bar{D}}_{d}^{2}=d_{i}] & =\frac{P\left[D_{d}^{2}=d_{i}\right]P_{1}\left(0,d_{i}-1,p\right)}{\sum_{d_{i}=k_{m}}^{n}P\left[D_{d}^{2}=d_{i}\right]P_{1}\left(0,d_{i}-1,p\right)} \end{array}$$ (13)

$$\begin{array}{cc} P[Z_{d}^{2}=x] & =\sum_{d_{i}=k_{m}}^{n}P\left[{\bar{D}}_{d}^{2}=d_{i}\right]P_{1}(x,d_{i}-1,1-\sum_{d_{i}=k_{m}}^{n}\frac{P\left[D=d_{i}\right]\;d_{i}}{\bar{k}}P_{1}\left(0,d_{i}-1,p\right)) \end{array}$$ (14)

$$\begin{array}{cc} P[W_{d}^{2}=x] & =\sum_{i=0}^{n}P\left[Z_{d}^{2}=i\right]P_{2}(x,\frac{1}{i+1}) \end{array}$$ (15)

Similarly to *F*_1*d*_(*x*), note that *F*_2*d*_(*x*) can be derived, if in addition to *p* and the pmf of $D_{d}^{1}$, the pmf of $D_{2}^{2}$ is known. Let *F*_*d*_(*x*) = *P*[*X*_*d*_ = *x*] represent the pmf of *X*_*d*_. The following result characterizes *F*_*d*_(*x*).

**Theorem 3**. The pmf of *X*_*d*_ is given by

$$\begin{array}{c} F_{d}\left(x\right)=\sum_{i=0}^{d}F_{1d}\left(i\right)F_{2d}^{i}\left(x-i-1\right) \end{array}$$ (16)

where

$$\begin{array}{cc} F_{2d}^{0}\left(x\right) & =\{\begin{array}{c} 1\;x=0\\ 0\;\text{otherwise} \end{array} \end{array}$$ (17)

$$\begin{array}{cc} F_{2d}^{1}\left(x\right) & =F_{2d}\left(x\right) \end{array}$$ (18)

$$\begin{array}{cc} F_{2d}^{i}\left(x\right) & =\sum_{m=-\infty }^{\infty }F_{2d}^{i-1}\left(x-m\right)F_{2d}\left(m\right) \end{array}$$ (19)

Note that if *F*_2*d*_ is known, then $F_{2d}^{i}$ can be obtained recursively. Let *F*(*x*) = *P*[*X* = *x*] represent the pmf of the sizes of the Voronoi cells in the case where generator nodes (events) are uniformly distributed. Note that

$$\begin{array}{c} F\left(x\right)=\sum_{d=k_{m}}^{n}P\left[D_{g}=d\right]F_{d}\left(x\right) \end{array}$$ (20)

Based on Theorems 1-3, we can now compute *F*(*x*) using the following algorithm.

**Algorithm 1** Computing the theoretical pmf of *X*.

**Input**: Pmfs of *D* and $D_{1}^{d}$, and *p*.

**Output**: *F*(*x*)

1: *k*_*m*_ ← minimum degree of all nodes in *G*

2: **for** *d* ← *k*_*m*_ to *n* **do**

3:  Compute the pmf of $R_{d}^{1},Z_{d}^{1}$, $W_{d}^{1}$, and *F*_1*d*_ (using Eqs (3)–(6))

4:  Approximate the pmf of $D_{d}^{2}$ (using Eq (1))

5:  Compute the pmf of $R_{d}^{1},G_{d}^{2}$, $Y_{d}^{2}$, ${\bar{D}}_{d}^{2}$, $Z_{d}^{2}$, $W_{d}^{2}$, and *F*_2*d*_ (using Eqs (9)–(15))

6:  Compute $F_{2d}^{0}$ and $F_{2d}^{1}$ (using Eqs (17) and (18))

7:  $F_{d}\left(x\right)=F_{1d}\left(0\right)F_{2d}^{0}\left(x-1\right)+F_{1d}\left(1\right)F_{2d}^{1}\left(x-2\right)$

8:  **for** *j* ← 2 to *d* **do**

9:   Compute $F_{2d}^{j}$ (using Eq (19))

10:   $F_{d}\left(x\right)\leftarrow F_{d}\left(x\right)+F_{1d}\left(j\right)F_{2d}^{j}\left(x-j-1\right)$

11:  **end for**

12: **end for**

13: Compute *F* (using Eq (20))

14: **return** *F*(*x*)

Algorithm 1 enables us to define the following criterion for determining whether the distribution of events in a network obeys a non-uniform distribution.

**Criterion 1 (Hotspot criterion)**. *Let F represent the pmf of the sizes of Voronoi cells when generator nodes are uniformly distributed and F*_*e*_ *the empirical pmf of the sizes of Voronoi cells for a given network*.

1. *For regular or Poisson networks, there is event concentration if*
  $$\begin{array}{c} \chi^{2}\left(F_{e},F\right)\geq c\left(\alpha \right) \end{array}$$ (21)
  *where c* > 0 *is a threshold* (*which determines the significance level α of the Chi Square distribution χ*^2^).
2. *For power law networks, there is event concentration if*
  $$\begin{array}{c} \Psi \left(Fe,F\right)=\frac{\bar{n}\left(F\right)-\bar{n}\left(F_{e}\right)}{\bar{n}\left(F\right)-1}\geq \beta \end{array}$$ (22)
  *where β* > 0 *represents a threshold*, *and*
  $$\begin{array}{cc} \bar{n}\left(F\right) & =4((\sum_{i=1}^{Q_{1}\left(F\right)-1}F\left(i\right)i)+(\frac{1}{4}-\sum_{i=1}^{Q_{1}\left(F\right)-1}F\left(i\right))Q_{1}\left(F\right)) \end{array}$$ (23)
  *represents the average size of the cells in the first quartile* (*Q*_1_(*F*)) *of F*.

Note that the criterion for identifying hotspots depends on the distribution of the sizes of the Voronoi cells, which in turn depends on the degree distribution of the network. The criterion compares the output distribution of Algorithm 1 with the empirical distributions of the sizes of Voronoi cells. Deviations from *F* indicate the amount of concentration of events on the network. For regular and Poisson networks, deviations are measured using the *χ*^2^ test. For power law networks, deviations are measured based on the average size of the cells in the first quartiles of *F* and *F*_*e*_.

## Empirical networks

### Chicago street network

We use data between January 1 and December 31, 2017, to evaluate the formation of assault hotspots on the street network of the city of Chicago [13]. The street network considers expressways, collectors, and arterials. It has 2902 edges, which represent streets, and 1650 nodes, which represent street intersections. An assault is represented as an event and associated to the nearest intersection. We first consider only handgun assaults and then widen the analysis for all types of assaults reported in the 12 months.

In particular, we evaluate the dispersion of assaults over time based on Criterion 1. To identify the length of the observation period that is required for a stationary, high-concentration outcome to be observed, we consider the following steps.

1. Define an observation period of *t* days, *t* = 7, 14, 21, ….
2. Consider assaults reported within *t* days after January 1, 2017.
3. Associate each reported assault to the closest street intersection (node) and mark each node with *ε* = 1 or more assaults as a generator node.
4. Apply Criterion 1 to determine if there is a concentration of events (i.e., evaluate whether generator nodes create a significant number of small, adjacent Voronoi cells).
5. Move the observation period by one day and repeat steps 1-4 (i.e, consider the assaults reported within *t* days after January 2, 2017).
6. Repeat steps 1-5 until the observation period starts on December 31, 2017.
7. For each observation period of length *t*, calculate the percentage of instances (throughout 2017) where the proposed criterion determines a high event concentration (that is, the percentage of instances that the null hypothesis of there being no hotspot formation is rejected).

Since the street network of Chicago resembles a lattice, we need to consider Criterion 1.1, that is, the criterion for detecting hotspot on a regular network. The average local clustering coefficient is 0.07 (a negligible value close to 0), meaning that there are hardly any connections in the network neighborhoods of any node. However, note that for a street network, nodes in *N*_2_ share two instead of a single neighbor in *N*_1_. As a consequence, condition 4 of Assumption 1 is not satisfied, and the derivation of the random variable *X*_*d*_ (Eq (2)) is no longer a precise expression. In particular, note that, if nodes in *N*_2_ share an additional neighbor, then the first term in Eq (2) will take into account some nodes at level 2 twice. Under such a scenario, the expression for *X*_*d*_ is hard to compute and an important part of our current research efforts. Nonetheless, Eq 2 serves as an approximation for the size of the cell of a randomly selected generator node with degree *d*. Accordingly, Algorithm 1 provides an approximation rather than a precise expression for *F*. Finally, to minimize the number of false positives in detecting hotspots, we use a significance level of *α* = 10^−4^.

**Fig 4:**
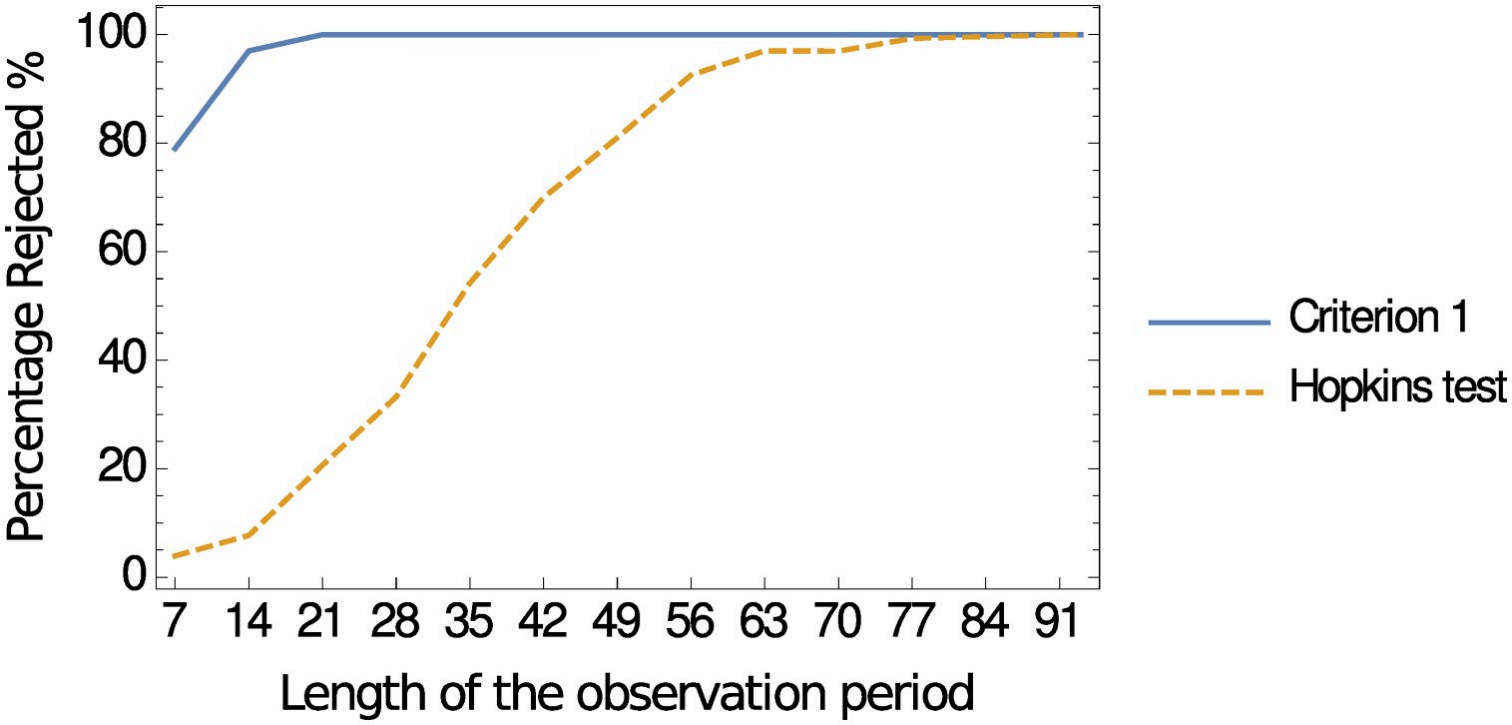
Percentage of instances that the null hypothesis is rejected for different observation periods of length *t* according to the proposed criterion (solid curve) and the Hopkins test (dashed curve).

The solid line in Fig 4 represents the percentage of instances that the null hypothesis is rejected for observation periods of different lengths. Note that the minimum period for which the criterion consistently identifies the formation of hotspots throughout 2017 is *t* = 21 days, in which case 100 percent of all 365 evaluations of the null hypothesis are rejected. That is, for an observation period of 21 days (or longer), the proposed criterion suggests that hotspots of handgun assaults are formed over the network. In contrast, the dashed curve in Fig 4 represents the percentage of instances that the null hypothesis is rejected based on the proximity of events in the metric space. In particular, it depicts the outcome of determining whether events are concentrated (i.e., step 4 above) when applying the Hopkins test [14] (instead of Criterion 1). Identifying the formation of hotspots for a Hopkins score below 0.25, the test requires an observation period of more than two months in order to reject the null hypothesis over 90 percent of all instances.

**Fig 5:**
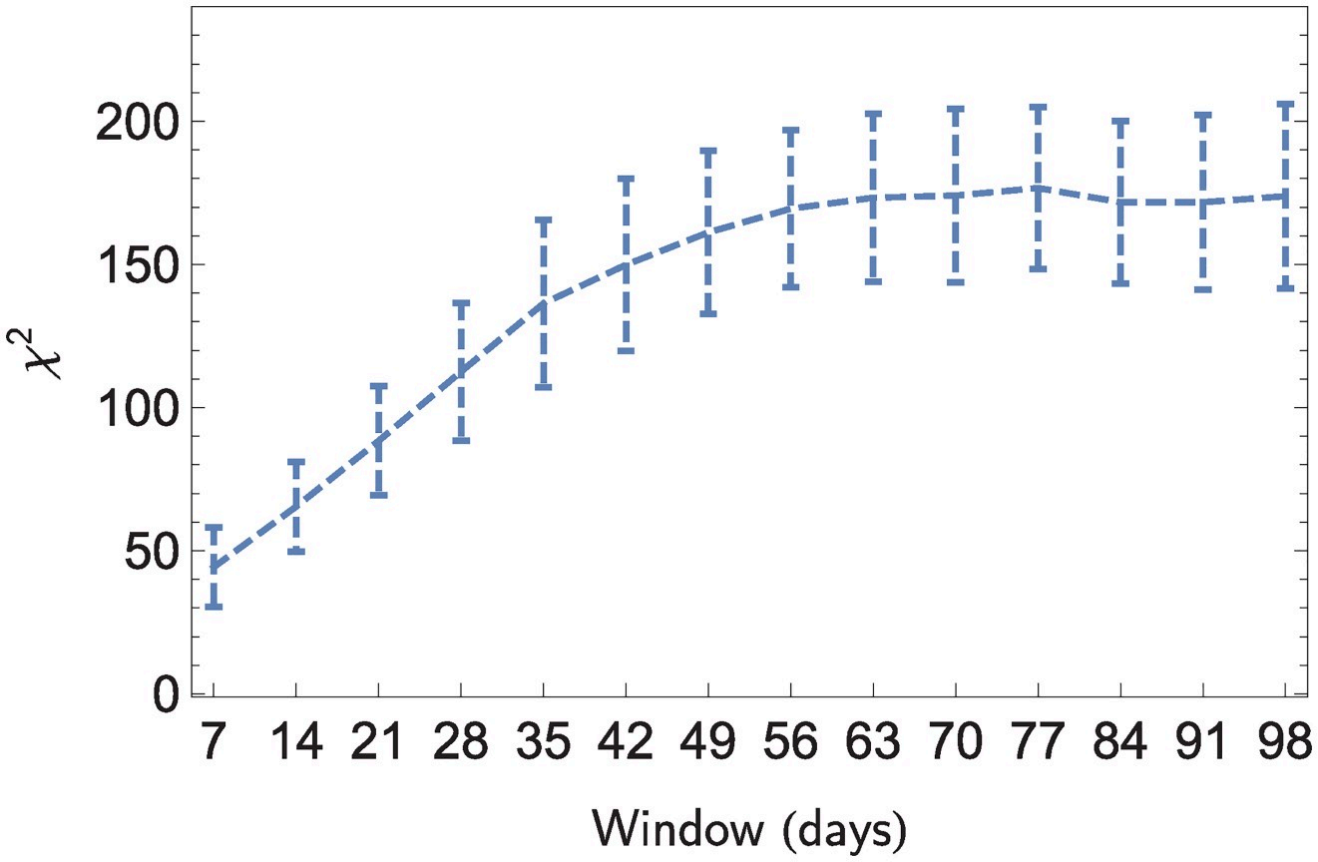
Value of *χ*^2^ test for different observation periods.

Next, Fig 5 shows the values of the *χ*^2^ test (Eq (21)) for different observation periods. The error bars represent one standard deviation. Note that for observation periods that are longer than two months, the values of *χ*^2^ remain approximately constant, meaning that the amount of event concentration on the network does not change significantly when longer observation periods are considered.

The analysis so far classifies as a generator node any intersection (node) associated on the street network to a single assault (event). That is, *ε* = 1. However, it is often of interest to distinguish between intersections where sporadic criminal activities take place and those where criminal activities are comparatively more frequent. We now evaluate the effect of classifying a generator node based on a stronger condition, that is, if at least a particular number of assaults (of any type) is associated to that node within a given observation period.

In particular, we revisit the procedure for determining the length of a period for a stationary, high-concentration outcome to be observed, and consider a varying event threshold *ε* ≥ 1 for different observations periods in step 3 above. As before, each reported assault is associated to the closest node in the street network. However, only nodes with a number of assaults of at least *ε* = *t*/7 are marked as generator nodes for observation periods of length *t* = 7, 14, 21, …. In other words, an intersection represents a generator node if, on average, more than one assault occurs every 7 days.

**Fig 6:**
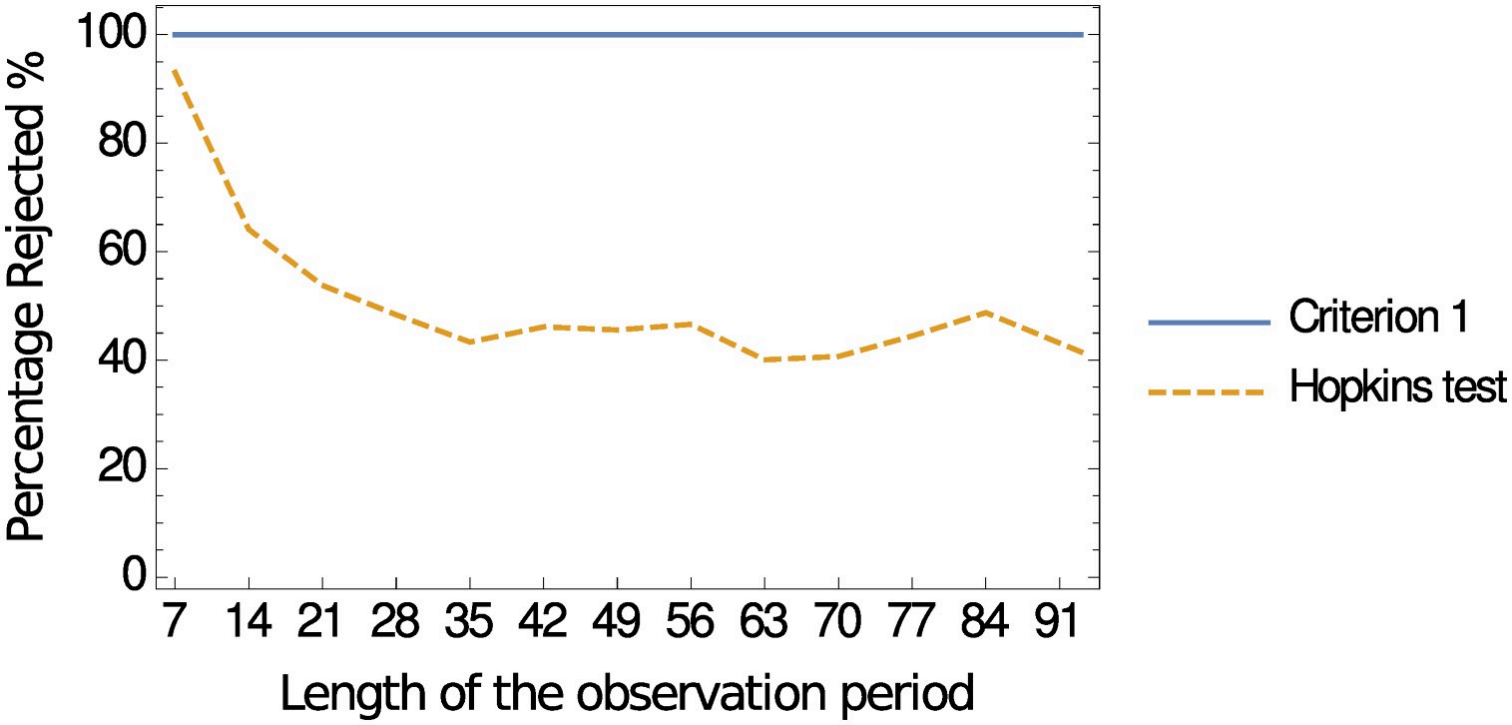
Percentage of instances that the null hypothesis is rejected for different observation periods of length *t* and varying event threshold *ε* = *t*/7 according to the proposed criterion (solid curve) and the Hopkins test (dashed curve).

Note that defining an event threshold *ε* that varies depending on the length of the observation period represents a stronger condition for classifying generator nodes. Step 3 above marks fewer nodes as generator nodes (compared to the case where *ε* = 1), since fewer intersections have a persistent high rate of assaults. Nonetheless, the solid line in Fig 6 shows that Criterion 1 identifies the formation of hotspots for observation periods of any length. The horizontal line indicates that, regardless of the length of the observation period, hotspots on the street network are formed as the outcome of high rates of assaults at intersections which are located relatively close to each other. The dashed line in Fig 6, in contrast, shows that when applying the Hopkins test for the same observation periods, the percentage of instances the null hypothesis is rejected (i.e, hotspot are detection) tends to decrease as longer periods are considered. According the Hopkins test, intersections with high rate of assaults are not close to each other in the metric space. Unlike to the proposed approach, the Hopkins test is not a decisive approach to identify a high concentration of events on the network (it now rejects the null hypothesis only about 40-50% of all instances).

### Co-purchase network of Amazon

Next, consider a co-purchase network of products from Amazon [15]. After a purchase, users can rate their satisfaction with the product they bought on a scale from 1 (very dissatisfied) to 5 (very satisfied). The co-purchase network consists of nodes that represent users and edges that connect users who bought the same product. The average rating of a user indicates overall user satisfaction. Dissatisfied users are defined as the users whose average rating is below to the 10^th^ percentile. Analyzing event concentration on the user co-purchase network enables us to evaluate whether dissatisfied users, who purchase a shared set of products, are concentrated on some parts of the network. The network contains 8444 nodes and 38492 edges.

**Fig 7:**
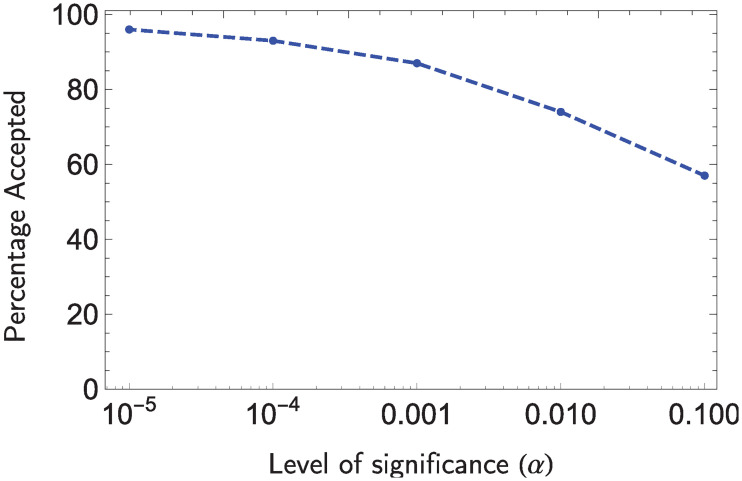
Percentage of instances that the the null hypothesis is accepted when generator nodes are located uniformly at random for 100 simulations.

The average local clustering of the network is 0.68, which implies that the network does not satisfy Assumption 1. Fig 7 assesses the quality of the approximation provided by Algorithm 1. It shows the percentage of simulations where the null hypothesis is accepted based on the Chi Square test for different significance levels *α*. Note that with *α* = 10^−4^ the null hypothesis is accepted more than 90% of all simulations. In other words, for a significance level *α* ≤ 10^−4^, the simulated distribution is equal to the theoretical approximation for more than the 90% of the runs. Though Assumption 1.3 is not satisfied, the approximation provided by Algorithm 1 is quite good.

**Fig 8:**
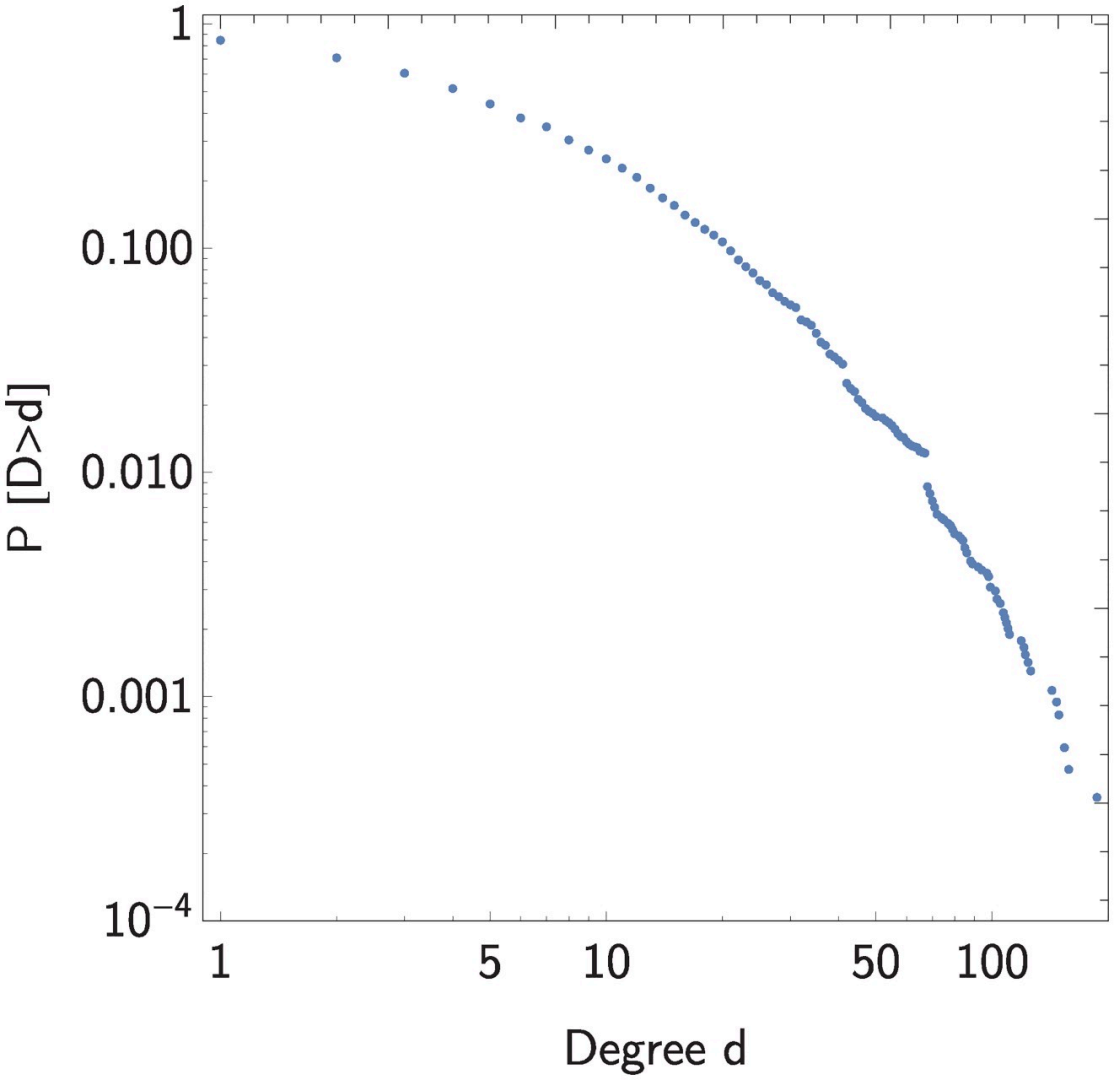
Complementary cumulative degree distribution of the co-purchase network of Amazon.

Fig 8 shows the complementary cumulative degree distribution. Given the power law that characterizes the tail of the degree distribution, we use Criterion 1.2 to determine whether events are concentrated.

**Fig 9:**
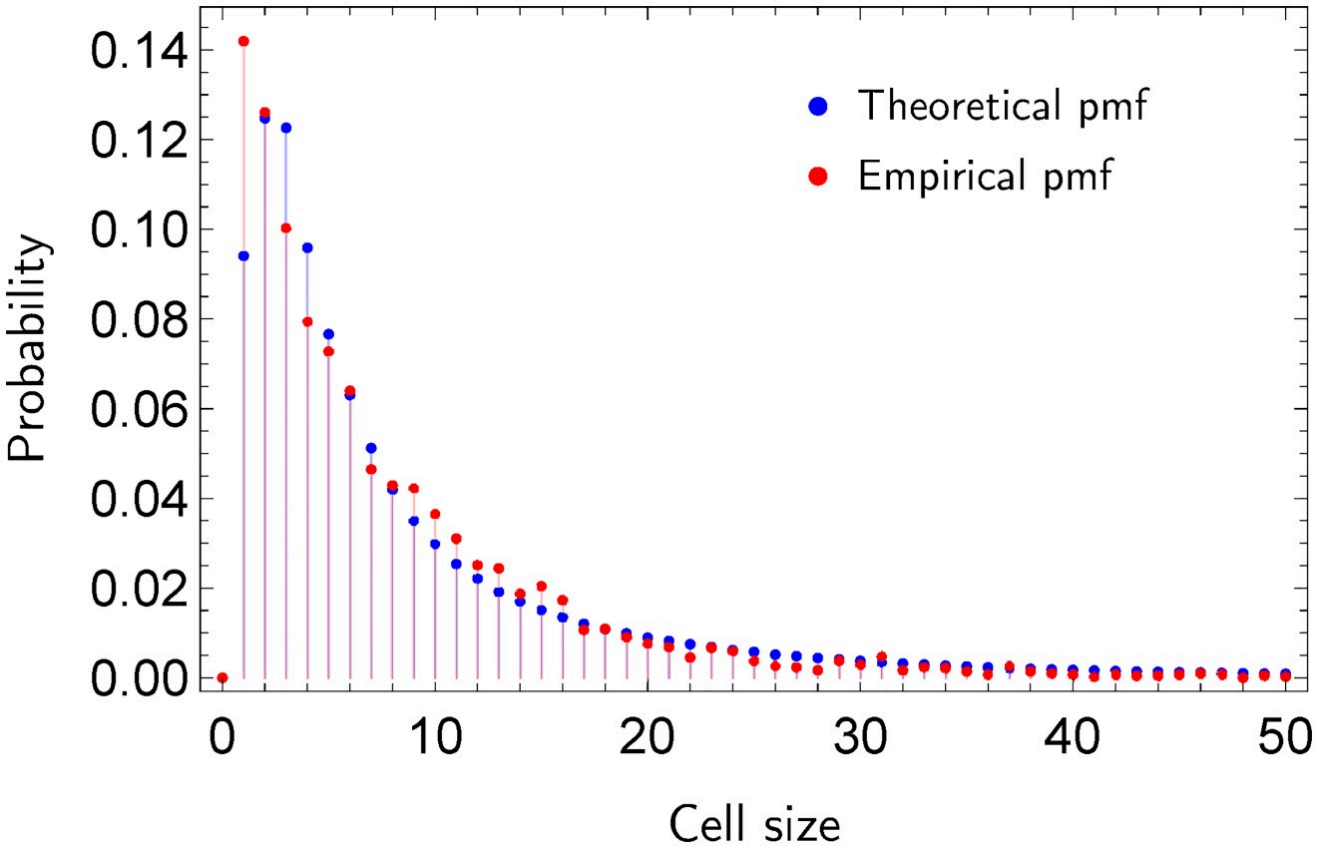
Distributions *F* and *F*_*e*_ when dissatisfied users are marked as generator nodes on the co-purchase network of Amazon.

Fig 9 shows the pmf of the distribution of the sizes of Voronoi cells and the theoretical distribution. The proposed criterion determines that dissatisfied users are not concentrated. Indeed, note that the empirical distribution resembles the theoretical distribution for which events are located uniformly at random. If we apply the *χ*^2^ test to both distributions, then the null hypothesis is accepted (for *α* = 10^−4^), which means that dissatisfied users are uniformly distributed across the network.

**Fig 10:**
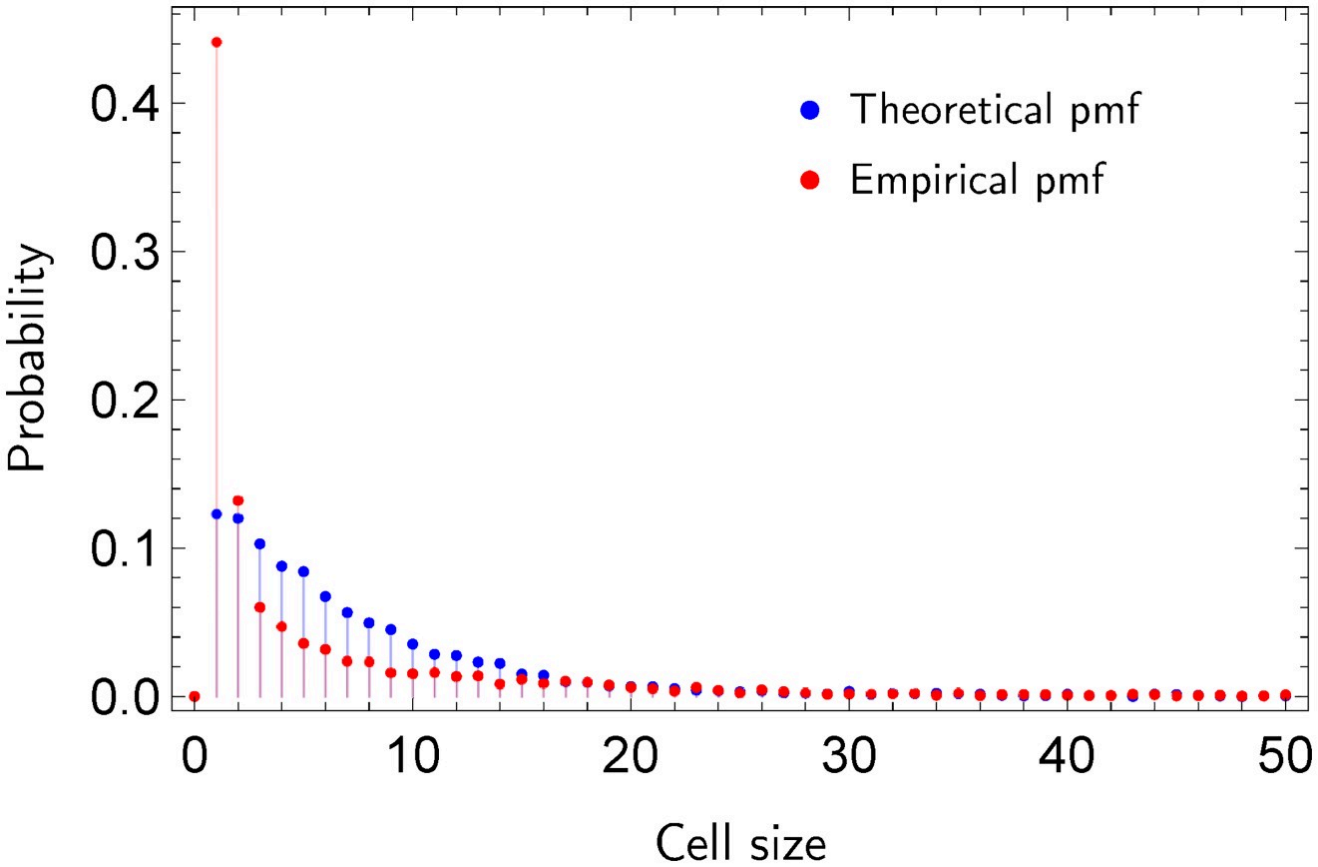
Distributions *F* and *F*_*e*_ when dissatisfied users are marked as events on the co-purchase network.

Finally, we modify the initial ratings to obtain an artificial concentration of dissatisfied users. To generate these hotspots, we first divide the co-purchase network into communities based on the measure of community modularity. Second, we select two communities such that the total number of members of both communities is approximately 844 (*p* = 0.1). Third, we select products that have been bought by at least two members of the two communities, and assign a rating of 1 to the transactions that involve these products. Finally, we compute the set of dissatisfied users based on the new average rating of each user. Note that dissatisfied users are now arbitrary concentrated across the two selected communities. Criterion 1.2 determines that dissatisfied users are now indeed concentrated. Fig 10 shows the pmf of the sizes of Voronoi cells when dissatisfied users are marked as events and the theoretical pmf from a uniform allocation. As expected, the number of cells of small size increases when events are concentrated. Note that the number of cells of size one in the empirical distribution is approximately four times larger than the number in the theoretical distribution.

## Discussion

The proposed framework enables us to derive a summary statistic for measuring event concentration based on Voronoi diagrams. It provides an approximation for the distribution of the sizes of Voronoi cells for regular, Poisson, and power law networks in which events are distributed uniformly at random. When the distribution of events obeys a non-uniform allocation, groups of small, adjacent Voronoi cells indicate subnetworks where events (generator nodes) are highly concentrated (hotspots are formed).

Building on this key property of Voronoi diagrams, the proposed criterion for detecting hotspots enables us to measure concentration across a variety of scenarios in which events are distributed over a network. Its applications range from determining whether events such as traffic accidents or fire outbreaks are concentrated in certain parts of a city, to evaluating whether influencers in a topic area (e.g., sports or politics) are gathered together in particular subgraphs of a social network.

Our work illustrates the criterion by analyzing the distribution of assaults on the street network of Chicago at various time scales, and considering various event thresholds *ε*. We show how the criterion can be used to estimate the smallest observation period for explaining the formation of stationary concentrations of events. Our analysis of the distribution of events over urban structures such as the street network aims to complement traditional approaches that identify clusters on the metric space. We compare the outcome of the proposed criterion to that of detecting hotspots by evaluating the proximity of events in the metric space (using the Hopkins test). Finally, we also measure event concentration in a co-purchase network and show that dissatisfied users are uniformly distributed over the network.

The results presented in this paper should be considered in the light of some limitations. The theoretical framework used to derive the hotspot criterion requires certain assumptions on the topological properties of the network, which are generally only approximately met by empirical networks. In particular, we assume that (i) the average local clustering coefficient is negligible (below 0.1), and (ii) the degree distribution of the nodes with events resembles that of the entire network. Satisfying these assumptions guarantees that Algorithm 1 can compute the pmf of the sizes of the cells for a network with a uniform event distribution. Analyzing the behavior of the proposed framework for networks with high clustering remains an interesting direction for future research.

## Appendix

### A: Proof of Theorem 1

**Theorem 1**. The pmf of $X_{d}^{1}$ is given by

$$\begin{array}{c} F_{1d}\left(x\right)=\sum_{i=0}^{d}P\left[R_{d}^{1}=i\right]P_{1}\left(x,i,P\left[W_{d}^{1}=1\right]\right) \end{array}$$ (3)

where

$$\begin{array}{cc} P[R_{d}^{1}=x] & =P_{1}\left(x,d,1-p\right) \end{array}$$ (4)

$$\begin{array}{cc} P[Z_{d}^{1}=x] & =\sum_{d_{i}=k_{m}}^{n}P\left[D_{d}^{1}=d_{i}\right]P_{1}(x,d_{i}-1,p) \end{array}$$ (5)

$$\begin{array}{cc} P[W_{d}^{1}=x] & =\sum_{i=0}^{n}P\left[Z_{d}^{1}=i\right]P_{2}(x,\frac{1}{1+i}) \end{array}$$ (6)

$$\begin{array}{cc} P_{1}\left(x,d,q\right) & =\left(\begin{array}{c} d\\ x \end{array}\right)q^{x}{\left(1-q\right)}^{d-x} \end{array}$$ (7)

$$\begin{array}{cc} P_{2}\left(x,q\right) & =q^{x}{\left(1-q\right)}^{1-x} \end{array}$$ (8)

*Proof*. To define the pmf of $X_{d}^{1}$, consider both regular and generator nodes in *N*_1_. Let $R_{d}^{1}$ be a random variable that denotes the number of regular nodes in *N*_1_. Since *p* is the probability of randomly selecting a generator node, we know that

$$\begin{array}{c} R_{d}^{1}∼\text{Bin}\left(d,1-p\right) \end{array}$$ (24)

**Remark 1**. *Based on definition 1*, *if a regular node v*_*i*_ *satisfies that ρ*(*v*_*i*_, *u*) ≤ *ρ*(*v*_*i*_, *u*_*j*_) *for all u*_*j*_ ∈ *U*, *and i* = |{*u*_*i*_ ∈ *U* \ {*u*}:*ρ*(*v*_*i*_, *u*_*i*_) = *ρ*(*v*_*i*_, *u*)}|, *then the probability that node v*_*i*_ *belongs to V*(*u*) *is* $\frac{1}{i+1}$. *Otherwise, the probability that v*_*i*_ *belongs to V*(*u*) *is 0*.

Note that a regular node in *N*_1_ does not necessarily belongs to *V*(*u*). According to remark 1, the probability that a regular node in *N*_1_ belongs to *V*(*u*) depends on the number of neighboring generator nodes of that node. Let $Z_{d}^{1}$ be a random variable that represents the number of generator nodes in *N*_2_, that are neighbors of a regular node in *N*_1_.

**Remark 2**. *Note that the distribution of the number of generator neighbors in N*_*δ*+1_ *of a regular node with degree d*_*i*_, *located in N*_*δ*_, *obeys a binomial distribution* Bin(*d*_*i*_ − 1, *p*).

According to Remark 2, the pmf of $Z_{d}^{1}$ is a mixture of binomial distributions

$$\begin{array}{c} P\left[Z_{d}^{1}=x\right]=\sum_{d_{i}=k_{m}}^{n}P\left[D_{d}^{1}=d_{i}\right]P_{1}(x,d_{i}-1,p) \end{array}$$ (25)

where

$$\begin{array}{c} P_{1}\left(x,d,q\right)≔\left(\begin{array}{c} d\\ x \end{array}\right)q^{x}{\left(1-q\right)}^{d-x} \end{array}$$

represents the pmf of the Binomial distribution.

Let $W_{d}^{1}$ be a Bernoulli random variable that indicates if a regular node in *N*_1_ belongs to *V*(*u*). According to Remark 1, note that

$$\begin{array}{c} P\left[W_{d}^{1}=x\right]=\sum_{i=0}^{n}P\left[Z_{d}^{1}=i\right]P_{2}(x,\frac{1}{1+i}) \end{array}$$ (26)

where

$$\begin{array}{c} P_{2}\left(x,q\right)≔q^{x}{\left(1-q\right)}^{1-x} \end{array}$$

represents the pmf of the Bernoulli distribution.

Note that $P[W_{d}^{1}=1]$ denotes the probability that a regular node in *N*_1_ belongs to *V*(*u*). Moreover, if there are a total of *i* regular nodes in *N*_1_, then $\text{Bin}(i,P\left[W_{d}^{1}=1\right])$ is the distribution of the number of nodes that belong to the cell in *N*_1_. The distribution of $X_{d}^{1}$ obeys

$$\begin{array}{c} F_{1d}\left(x\right)=\sum_{i=0}^{d}P\left[R_{d}^{1}=i\right]P_{1}\left(x,i,P\left[W_{d}^{1}=1\right]\right) \end{array}$$ (27)

### B: Proof of Theorem 2

**Theorem 2**. The pmf of $X_{d}^{2i}$ is given by

$$\begin{array}{c} F_{2d}\left(x\right)=\sum_{i=0}^{n}P\left[Y_{d}^{2}=i\right]P_{1}\left(x,i,P\left[W_{d}^{2}=1\right]\right) \end{array}$$ (9)

where

$$\begin{array}{cc} P[R_{d}^{2}=x] & =\frac{\sum_{d_{j}=i+1}^{n}P\left[D_{d}^{1}=d_{j}\right]P_{1}\left(x,d_{j}-1,1-p\right)(\frac{1}{d_{j}-i})}{\sum_{k=0}^{n}\sum_{d_{j}=k+1}^{n}P\left[D_{d}^{1}=d_{j}\right]P_{1}\left(k,d_{j}-1,1-p\right)(\frac{1}{d_{j}-k})}, \end{array}$$ (10)

$$\begin{array}{cc} P[G_{d}^{2}=x] & \approx \sum_{d_{i}=k_{m}}^{n}\frac{P\left[D=d_{i}\right]\;d_{i}}{\bar{k}}P_{1}\left(x,d_{i}-1,p\right) \end{array}$$ (11)

$$\begin{array}{cc} P[Y_{d}^{2}=x] & =\sum_{i=0}^{n}P\left[R_{d}^{2}=i\right]P_{1}\left(x,i,P\left[G_{d}^{2}=0\right]\right) \end{array}$$ (12)

$$\begin{array}{cc} P[{\bar{D}}_{d}^{2}=d_{i}] & =\frac{P\left[D_{d}^{2}=d_{i}\right]P_{1}\left(0,d_{i}-1,p\right)}{\sum_{d_{i}=k_{m}}^{n}P\left[D_{d}^{2}=d_{i}\right]P_{1}\left(0,d_{i}-1,p\right)} \end{array}$$ (13)

$$\begin{array}{cc} P[Z_{d}^{2}=x] & =\sum_{d_{i}=k_{m}}^{n}P\left[{\bar{D}}_{d}^{2}=d_{i}\right]P_{1}(x,d_{i}-1,1-\sum_{d_{i}=k_{m}}^{n}\frac{P\left[D=d_{i}\right]\;d_{i}}{\bar{k}}P_{1}\left(0,d_{i}-1,p\right)) \end{array}$$ (14)

$$\begin{array}{cc} P[W_{d}^{2}=x] & =\sum_{i=0}^{n}P\left[Z_{d}^{2}=i\right]P_{2}(x,\frac{1}{i+1}) \end{array}$$ (15)

*Proof*. We extend the analysis described in the appendix A: Proof of Theorem 1 of *N*_1_ to *N*_2_. Let $R_{d}^{2}$ denote a random variable that represents the number of regular nodes, located in *N*_2_, that are neighbors of a single node in *V*(*u*) ∩ *N*_1_. Note that a node with degree *d_j_* in *N*_1_ has, with probability *P*_1_(*i*, *d_j_* − 1, 1 − *p*), a total of *i* regular neighbors in *N*_2_. Based on Remark 1, this node belong to *V*(*u*) with probability $\frac{1}{d_{j}-i}$. The probability that a node that belongs to *V*(*u*), located in *N*_1_, has *i* regular nodes in *N*_2_ is given by

$$\begin{array}{c} P\left[R_{d}^{2}=x\right]=\frac{\sum_{d_{j}=i+1}^{n}P\left[D_{d}^{1}=d_{j}\right]P_{1}\left(x,d_{j}-1,1-p\right)(\frac{1}{d_{j}-i})}{\sum_{k=0}^{n}\sum_{d_{j}=k+1}^{n}P\left[D_{d}^{1}=d_{j}\right]P_{1}\left(k,d_{j}-1,1-p\right)(\frac{1}{d_{j}-k})} \end{array}$$ (28)

**Fig 11:**
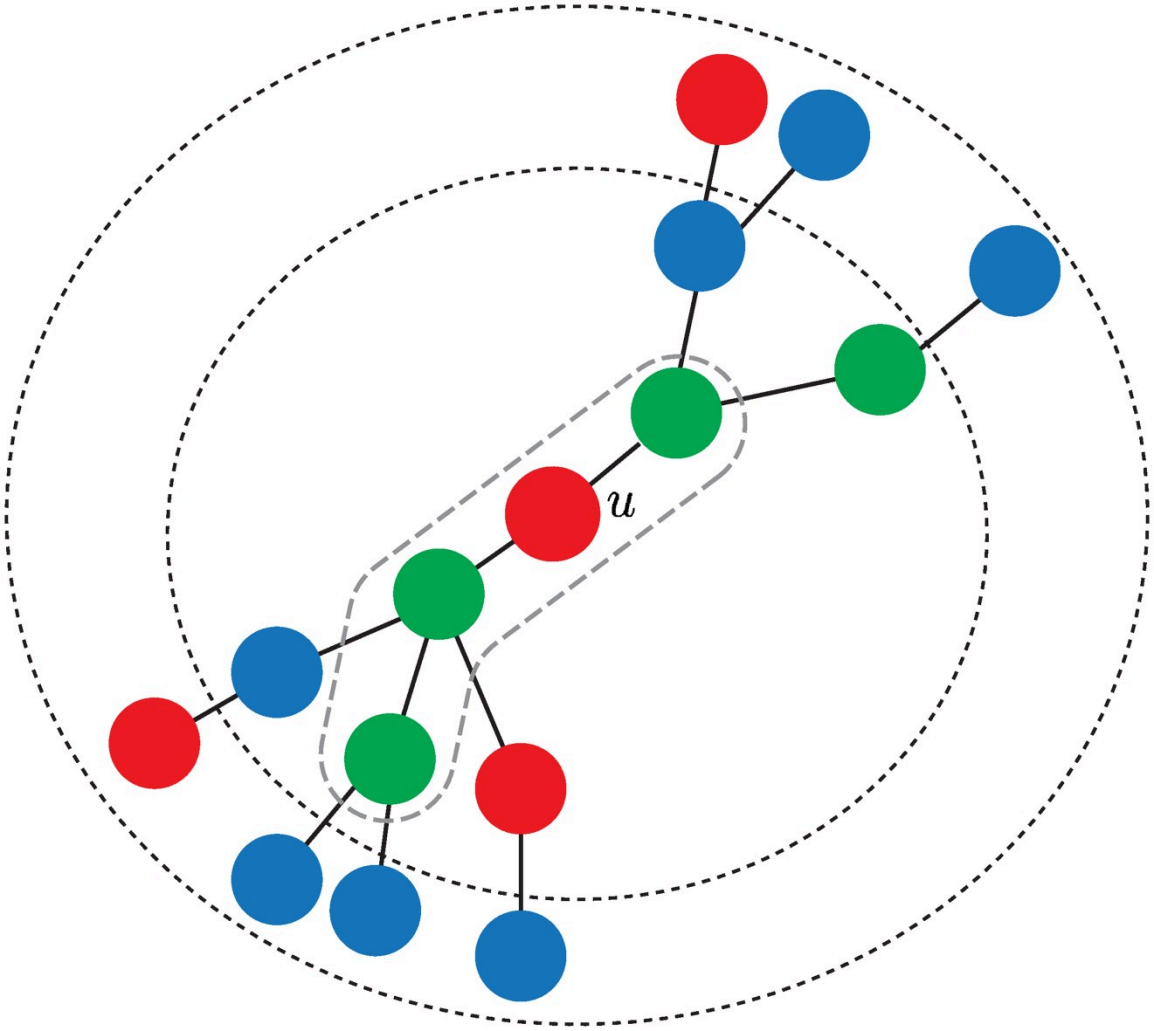
The concept of candidate node. Generator nodes are marked in red, regular nodes in blue, and candidates nodes of *V*(*u*) in green.

Note that the probability that a regular node, located in *N*_1_ of node *u*, belongs to *V*(*u*) is greater than 0. However, according to Remark 1, this is not true for *N*_*δ*_ when *δ* ≥ 2. For instance, if a regular node, located in *N*_2_, has a neighboring generator node, located in *N*_3_, then the probability that the regular node belongs to *V*(*u*) is 0. We refer to a regular node that has a probability greater than 0 to belong to *V*(*u*), as a *candidate node*. In other words, a regular node *v* is a candidate node if for all *u*′ ∈ *U*: *ρ*(*v*, *u*) ≤ *ρ*(*v*, *u*′). Note that each regular node, located in *N*_1_, is a candidate node. Fig 11 highlights the candidates nodes in green.

Consider a randomly selected regular node *v*, located in *N*_2_, that is a neighbor of a single node in *V*(*u*) ∩ *N*_1_. Let $G_{d}^{2}$ denote a random variable that represents the number of neighboring generator nodes of *v*, located in *N*_3_. According to Remark 2 and using the approximation given in Eq (1), note that

$$\begin{array}{c} P\left[G_{d}^{2}=x\right]=\sum_{d_{i}=k_{m}}^{n}P\left[D_{d}^{2}=d_{i}\right]P_{1}\left(x,d_{i}-1,p\right)\approx \sum_{d_{i}=k_{m}}^{n}\frac{P\left[D=d_{i}\right]\;d_{i}}{\bar{k}}P_{1}\left(x,d_{i}-1,p\right) \end{array}$$ (29)

Furthermore, let $Y_{d}^{2}$ denote a random variable that represents the number of candidate nodes in *N*_2_ that are neighbors for a single node that belongs to *V*(*u*) ∩ *N*_1_. A regular node in *N*_2_ is a candidate node if there is not a neighboring generator node in *N*_3_. So the probability of a regular node in *N*_2_ of being a candidate node of *V*(*u*) is $P[G_{d}^{2}=0]$. Then, note that if a node *v*, located in *N*_1_, has *i* neighboring regular nodes, the distribution of the number of neighboring candidate nodes in *N*_2_ for node *v* obeys $\text{Bin}(i,P\left[G_{d}^{2}=0\right])$. That is

$$\begin{array}{c} P\left[Y_{d}^{2}=x\right]=\sum_{i=0}^{n}P\left[R_{d}^{2}=i\right]P_{1}\left(x,i,P\left[G_{d}^{2}=0\right]\right) \end{array}$$ (30)

Based on the distribution of candidate nodes in *N*_2_, we now calculate the distribution of the number of nodes that belong to *V*(*u*) ∩ *N*_2_. Let ${\bar{D}}_{d}^{2}$ denote a random variable that characterizes the degree of a randomly selected candidate node in *N*_2_. According to Remark 2, *P*_1_(0, *d*_*i*_−1, *p*) is the probability that a node with degree *d*_*i*_, located in *N*_2_, has no generator nodes as neighbors in *N*_3_. So the probability that a randomly selected candidate node in *N*_2_ has degree *d*_*i*_ is given by

$$\begin{array}{c} P\left[{\bar{D}}_{d}^{2}=d_{i}\right]=\frac{P\left[D_{d}^{2}=d_{i}\right]P_{1}\left(0,d_{i}-1,p\right)}{\sum_{d_{i}=k_{m}}^{n}P\left[D_{d}^{2}=d_{i}\right]P_{1}\left(0,d_{i}-1,p\right)} \end{array}$$ (31)

**Fig 12:**
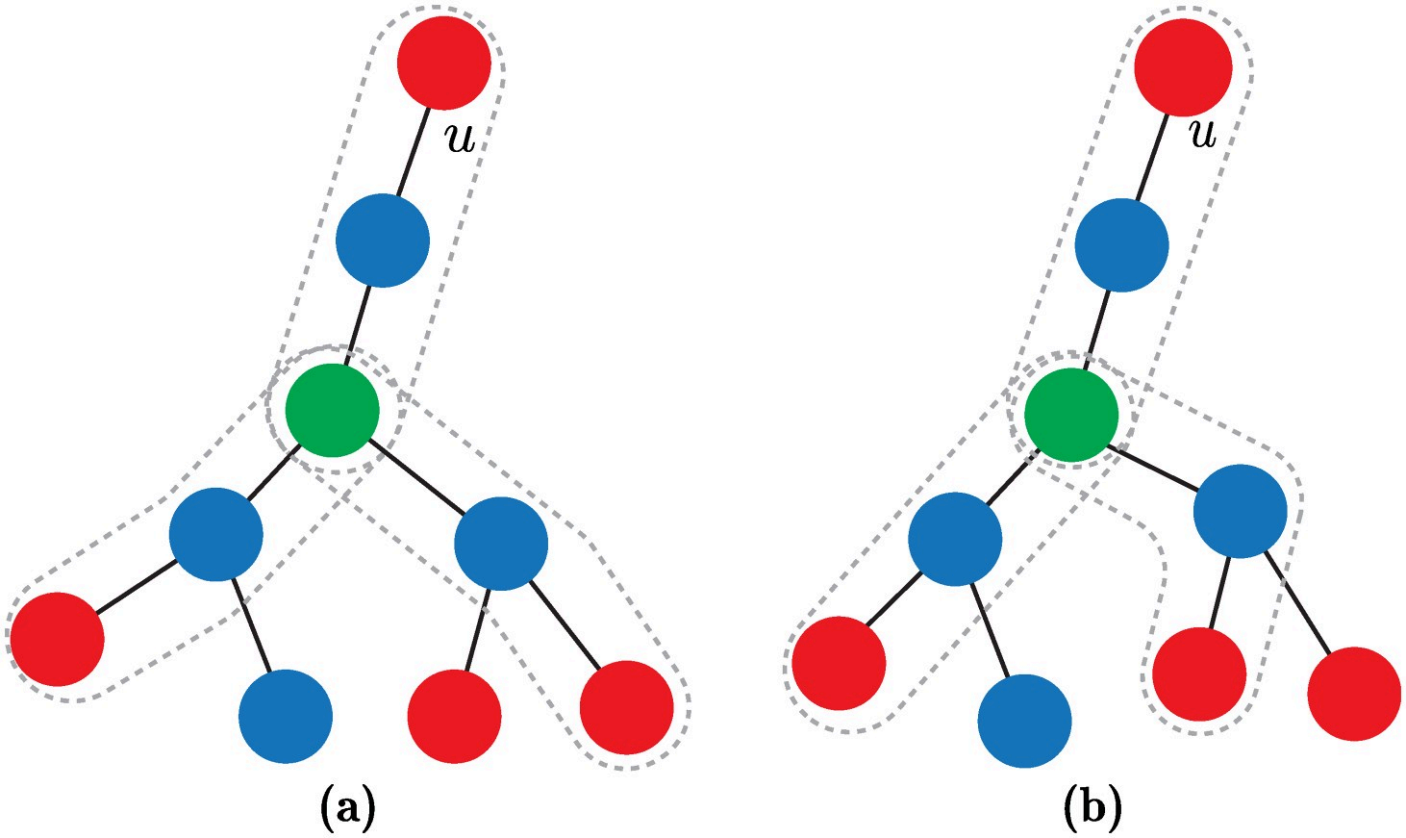
The concept of candidate node in *N*_2_. Representation of a candidate node of *V*(*u*) in *N*_2_ that can potentially belong to three different Voronoi cells.

Note that a candidate node in *N*_2_ satisfies that its distance to any generator node is greater or equal to 2. Let $Z_{d}^{2}$ denote a random variable that represents the number of generator nodes, except node *u*, that are at distance of 2 for a candidate node that is located in *N*_2_. In other words, $Z_{d}^{2}$ represents the number of Voronoi cells, except *V*(*u*), that can contain a candidate node, located in *N*_2_ of node *u*. Fig 12 depicts a candidate node of *V*(*u*) that can be potentially contained in three Voronoi cells including *V*(*u*), i.e., the probability that the candidate node belongs to any of the three cells is $\frac{1}{3}$. Note that for each regular node in *N*_3_ that has at least one neighboring generator in *N*_4_, there is an additional Voronoi cell that can potentially contain the candidate node in *N*_2_. Based on Eq (1), an approximation of the probability that a node in *N*_3_ has at least one neighboring generator node in *N*_4_ is

$$\begin{array}{c} 1-\sum_{d_{i}=k_{m}}^{n}\frac{P\left[D=d_{i}\right]\;d_{i}}{\bar{k}}P_{1}\left(0,d_{i}-1,p\right) \end{array}$$

Furthermore, note that the distribution of the number of generator nodes, except node *u*, that can contain in their cells a candidate node in *N*_2_ with degree *d*_*i*_ is given by

$$\begin{array}{c} \text{Bin}(d_{i}-1,1-\sum_{d_{i}=k_{m}}^{n}\frac{P\left[D=d_{i}\right]\;d_{i}}{\bar{k}}P_{1}\left(0,d_{i}-1,p\right)) \end{array}$$

According to Remark 1, the pmf of $Z_{d}^{2}$ is the mixture of binomial distributions

$$\begin{array}{c} P\left[Z_{d}^{2}=x\right]=\sum_{d_{i}=k_{m}}^{n}P\left[{\bar{D}}_{d}^{2}=d_{i}\right]P_{1}(x,d_{i}-1,1-\sum_{d_{i}=k_{m}}^{n}\frac{P\left[D=d_{i}\right]\;d_{i}}{\bar{k}}P_{1}\left(0,d_{i}-1,p\right)) \end{array}$$ (32)

If $W_{d}^{2}$ is a Bernoulli random variable that indicates the probability that a candidate node in *N*_2_ belongs to *V*(*u*), then

$$\begin{array}{c} P\left[W_{d}^{2}=x\right]=\sum_{i=0}^{n}P\left[Z_{d}^{2}=i\right]P_{2}(x,\frac{1}{i+1}) \end{array}$$ (33)

Based on the pmf of $W_{d}^{2}$, the distribution of $X_{d}^{2}$ is given by

$$\begin{array}{c} F_{2d}\left(x\right)=\sum_{i=0}^{n}P\left[Y_{d}^{2}=i\right]P_{1}\left(x,i,P\left[W_{d}^{2}=1\right]\right) \end{array}$$ (34)

### C: Proof of Theorem 3

**Theorem 3**. The pmf of *X*_*d*_ is given by

$$\begin{array}{c} F_{d}\left(x\right)=\sum_{i=0}^{d}F_{1d}\left(i\right)F_{2d}^{i}\left(x-i-1\right) \end{array}$$ (16)

where

$$\begin{array}{cc} F_{2d}^{0}\left(x\right) & =\{\begin{array}{c} 1\;x=0\\ 0\;\text{otherwise} \end{array} \end{array}$$ (17)

$$\begin{array}{cc} F_{2d}^{1}\left(x\right) & =F_{2d}\left(x\right) \end{array}$$ (18)

$$\begin{array}{cc} F_{2d}^{i}\left(x\right) & =\sum_{m=-\infty }^{\infty }F_{2d}^{i-1}\left(x-m\right)F_{2d}\left(m\right) \end{array}$$ (19)

*Proof*. To define the pmf of *X*_*d*_, recall that $P\left[X_{d}^{2i}=x\right]=F_{2d}\left(x\right)$. It can be shown that

$$\begin{array}{c} P\left[X_{d}^{21}+X_{d}^{22}=x\right]=\sum_{m=-\infty }^{\infty }F_{2d}\left(x-m\right)F_{2d}\left(m\right) \end{array}$$

Let $F_{2d}^{2}\left(x\right)=\sum_{m=-\infty }^{\infty }F_{2d}\left(x-m\right)F_{2d}\left(m\right)$. The distribution of the sum of multiple instances of a random variable can be generalized as

$$\begin{array}{c} P\left[\underset{j+1}{\underset{︸}{X_{d}^{21}+X_{d}^{22}+\cdots +X_{d}^{2(j+1)}}}=x\right]=\sum_{m=-\infty }^{\infty }F_{2d}^{j}\left(x-m\right)F_{2d}\left(m\right) \end{array}$$ (35)

where $F_{2d}^{j}\left(x\right)≔\sum_{m=-\infty }^{\infty }F_{2d}^{j-1}\left(x-m\right)F_{2d}\left(m\right)$. Based on Eqs (2) and (35), the pmf of *X*_*d*_ can be written as a mixture distribution

$$\begin{array}{c} F_{d}\left(x\right)≔P\left[X_{d}=x\right]=\sum_{i=0}^{d}F_{1d}\left(i\right)F_{2d}^{i}\left(x-i-1\right) \end{array}$$ (36)

where

$$\begin{array}{cc} F_{2d}^{0}\left(x\right) & =\{\begin{array}{c} 1\;x=0\\ 0\;\text{otherwise} \end{array} \end{array}$$ (37)

$$F_{2d}^{1}\left(x\right)=F_{2d}\left(x\right)$$ (38)

The first term in Eq (36) indicates the probability that *i* nodes in *N*_1_ belong to *V*(*u*). The second term represents the pmf of the sum of *i* instances of $X_{d}^{2}$, with a shift of *i* + 1 to account for the number of nodes in *N*_1_ and for the generator node. Note that if the number of nodes in *N*_1_ that belong to *V*(*u*) is zero, then it is not possible that a node in *N*_2_ belongs to *V*(*u*), according to Eq (37).
